# Behavioral Modeling and its Association with PrEP and ART Use in Ugandan HIV-Serodifferent Couples

**DOI:** 10.1007/s10461-024-04286-2

**Published:** 2024-02-16

**Authors:** Liying Wang, Timothy R. Muwonge, Jane M. Simoni, Florence Nambi, Lylianne Nakabugo, Joseph Kibuuka, Dorothy Thomas, Ingrid T. Katz, Erika Feutz, Katherine K. Thomas, Norma C. Ware, Monique A. Wyatt, Herbert Kadama, Andrew Mujugira, Renee Heffron, For the Partners PrEP Program Study Team

**Affiliations:** 1 Department of Psychology, University of Washington, Seattle, WA USA; 2 Infectious Diseases Institute, Makerere University, Kampala, Uganda; 3 Department of Global Health, University of Washington, Seattle, WA USA; 4 Present Address: Behavioral and Social Sciences Research, NIH, Bethesda, Maryland USA; 5 Office of Behavioral and Social Sciences Research (OBSSR), NIH, Bethesda, Maryland USA; 6 Division of Program Coordination, Planning and Strategic Initiatives (DPCPSI), NIH, Bethesda, Maryland USA; 7 National Institutes of Health (NIH), Bethesda, Maryland USA; 8 Department of Global Health and Social Medicine, Harvard Medical School, 25 Shattuck St, Boston, Massachusetts USA; 9 Department of Medicine, Brigham and Women’s Hospital, 75 Francis St, Boston, Massachusetts USA; 10 Harvard Global, Cambridge, MA USA; 11 Ministry of Health, Box 7272, Kampala, Uganda; 12 Department of Medicine, University of Alabama at Birmingham, 845 19th Street South Bevill Biomedical Research Building, Room 256D, Birmingham, AL 35294-2170 USA

**Keywords:** Observational learning, Behavioral modeling measure, HIV, Sero-different couple, Medication adherence

## Abstract

Integrating Pre-Exposure Prophylaxis (PrEP) delivery into Antiretroviral Therapy (ART) programs bridges the Human Immunodeficiency Virus (HIV) prevention gap for HIV-serodifferent couples prior to the partner living with HIV achieving viral suppression. Behavioral modeling is one mechanism that could explain health-related behavior among couples, including those using antiretroviral medications, but few tools exist to measure the extent to which behavior is modeled. Using a longitudinal observational design nested within a cluster randomized trial, this study examined the factor structure and assessed the internal consistency of a novel 24-item, four-point Likert-type scale to measure behavioral modeling and the association of behavioral modeling with medication-taking behaviors among heterosexual, cis-gender HIV-serodifferent couples. In 149 couples enrolled for research, a five-factor model provided the best statistical and conceptual fit, including attention to partner behavior, collective action, role modeling, motivation, and relationship quality. Behavioral modeling was associated with medication-taking behaviors among members of serodifferent couples. Partner modeling of ART/PrEP taking could be an important target for assessment and intervention in HIV prevention programs for HIV serodifferent couples.

**Supplementary Information:**

The online version contains supplementary material available at 10.1007/s10461-024-04286-2.

## Introduction

In the context of Human Immunodeficiency Virus (HIV) treatment, adherence to antiretroviral medication is an important health behavior that facilitates viral suppression, the driver of better health outcomes and prevention of onward HIV transmission. The sexual partnership within HIV-serodifferent couples, where one partner is living with HIV and the other is HIV-negative, puts the HIV-negative partner at high risk for HIV acquisition when adequate strategies for HIV prevention and treatment are not in place [1, 2]. Programs for HIV-serodifferent couples focus on supporting HIV treatment for the partner living with HIV and time-limited pre-exposure prophylaxis (PrEP) using antiretroviral medication for HIV-negative partners, when HIV viremia may not be suppressed in the partner living with HIV [3–5]. When PrEP is integrated into antiretroviral treatment (ART) programs for HIV-serodifferent couples, it bridges the immediate HIV prevention gap prior to viral suppression in the partner living with HIV and has been demonstrated to have 95% effectiveness in reducing HIV incidence [6]. A randomized trial of oral antiretroviral therapy for HIV-serodiscordant heterosexual couples in Kenya and Uganda found 67% and 75% reduction in the incidence of HIV with tenofovir and combination tenofovir–emtricitabine respectively [7]. The HIV-negative partners chose to take PrEP to reduce the HIV transmission anxiety, improve the relationship, and together build shared protection against HIV as a couple [8].

Behavioral modeling, or observational learning, refers to the process of individuals learning and acquiring new behaviors from watching others [9]. Observational learning has four processes, including attention, retention, production, and motivation. Individuals first direct their attention to the modeled behaviors or interactions (attention) and remember them (retention), which is dependent on individuals’ intellectual capacities. Individuals then perform the modeled behavior when the circumstances and physical skills allow them to (production). Motivation is the internal factor that drives the production of the modeled behavior, depending on the expectations about costs and benefits [7]. Within HIV-serodifferent couples using antiretrovirals for prevention and treatment, behavioral modeling is one mechanism that could explain each partner’s medication adherence behavior. Specifically, partners living with HIV could build self-efficacy for taking HIV medications by observing their HIV-negative partners manage medication side effects, adherence, and HIV-related stigma. The perceived benefits on their health, relationship quality, and shared future with their partners may also motivate ART adherence [10]. Observing the partners taking ART could reinforce PrEP adherence among HIV-negative partners in return, creating a positive feedback loop within the partners.

To our knowledge, no measure has been developed yet to capture whether heterosexual HIV-serodifferent couples exhibit behavioral modeling in their antiretroviral use (PrEP for prevention, ART for treatment). The current study aimed to (1) use factor analysis to identify the underlying factors that the items on a questionnaire are measuring and examine the internal consistency of a new behavioral modeling scale; (2) examine the association between behavioral modeling with medication-taking behaviors among HIV-serodifferent couples; and (3) explore the association between behavioral modeling with viral load levels among partners with HIV and Tenofovir (TFV) drug levels among HIV-negative partners.

## Methods

### Study Design and Participants

This study is a longitudinal observational study that was nested within the Partners PrEP Program, a stepped wedge cluster randomized trial evaluating the integration of PrEP delivery into ART programs (#NCT03586128) 11] in Kampala and Wakiso, Uganda from June 2018 to December 2021. Heterosexual sero-different couples were recruited into the program through couples-based HIV testing and received access to standard of care for HIV care after the intervention was launched at their clinic.

Participant inclusion criteria were as follows. For both members of the couple: (1) Age ≥ 18 years; (2) Ability and willingness to provide informed consent; (3) Being sexually active with each other; (4) Willingness to engage with the clinic system collaboratively. For the HIV-positive members (index participants): (1) Diagnosis as HIV-positive per the national HIV testing algorithm; (2) Recent identification as a member of an HIV serodiscordant couple; (3) Absence of current enrollment in any HIV treatment clinical trial. For the HIV-negative members (partner participants): (1) Diagnosis as HIV-negative using the national HIV testing algorithm; (2) Recent identification within an HIV serodiscordant relationship; (3) Absence of current enrollment in any HIV treatment clinical trial; (4) Non-usage of PrEP at present; (5) Eligibility for PrEP as defined by the WHO or Ugandan national guidelines [12].

Programmatic data were abstracted from patient medical charts including dates when PrEP and ART were dispensed and HIV viral load results from testing that occurred approximately 3–6 months after ART initiation in alignment with national guidelines. Additionally, couples were invited to enroll for supplemental research procedures that included interviewer-administered questionnaires on every quarter for about 24 months to ascertain data on demographics, sexual behavior, modeled behavior, and self-reported PrEP and ART adherence. A dried blood spot was also collected from HIV-negative partners in the research cohort and a random sample of 10% were selected for quantification of tenofovir (TFV)-diphosphate levels via validated liquid chromatography/tandem mass spectrometry methods [13].

### PrEP Delivery Program

The 12 ART clinics received training on PrEP delivery, ongoing technical assistance visits from the training team, and PrEP commodities before the program was launched. The PrEP training protocol was adapted from the national PrEP training curriculum in Uganda, with adjustments to tailor the service to HIV-serodifferent couples. The technical assistance visits were conducted by the training team who were experienced in PrEP delivery and clinical management. Once clinics entered the PrEP delivery stage, trained staff offered PrEP to the HIV-negative members of recently diagnosed HIV-serodifferent couples [14]. HIV-negative partners were counseled to use PrEP for at least 6 months after their partners started ART and supported to discontinue when the partner sustained ART use and there were no other potential sources of exposure (e.g., other partners).

### Measurements

#### Behavioral Modeling Scale

Through discussions and initial pilot testing with a group of clinicians, ART clients and their partners, and research staff, we developed a 28-item questionnaire with a four-point Likert-type response scale to measure behavioral modeling. The items were created based on the observational learning concepts of the Social Cognitive Theory [7]. Nineteen items mapped to the four processes of observational learning: attention (e.g., “I often see my partner taking PrEP/ART”), retention (e.g., “I remind my partner to take PrEP”), production (e.g., “I take my ART at the same time my partner takes PrEP/ART”), and motivation (e.g., “When my partner takes PrEP/ART I have hope for our future together”) [9]. Additionally, we added 9 items to capture the relationship quality between the partners (e.g., “I feel close to my partner”). These items were based on previous research that suggested a strong association between relationship satisfaction and PrEP adherence, and that role modeling is more effective when it comes from someone the observer feels close to and values [15]. Four items were removed due to poor face validity. Face validity refers to whether a question appears to measure the construct it aims to measure [16]. For example, one item that was removed was “I have people I can talk to about my relationship when I need support”, which was more about social support than behavioral modeling.

The final scale contained 24 items. The items were identical for both partners within a couple, with “PrEP” or “ART” used respectively for the HIV-negative partners and partners living with HIV. The response options ranged from (0) “Strongly disagree” to (3) “Strongly agree”. The scale was scored by computing a total raw score ranging from 0 to 72. Separate total scores were computed once subscales were identified. We computed total scores for both HIV-negative partners and the partners living with HIV to capture each partner’s self-report of the other partners’ behaviors that could have been observed.

#### Self-Reported ART/PrEP-Taking Behavior

Medication -aking behaviors were measured using three self-reported items adapted from Wilson et al. (1) whether the partner has taken ART/PrEP since the last clinic visit (yes/no); (2) how well the client took their ART/PrEP medication as directed in the past month (0 = very poor to 5 = excellent), and (3) how often the client took their ART/PrEP medication in the past month (0 = not at all to 3 = every day). We performed linear transformation on the item responses for the second and the third item to compose the adherence score (with a range 0-100), with higher scores indicating better adherence [17].

### Statistical Analysis

All analyses were conducted in R [18]. Demographic characteristics of the sample and medication-taking behaviors across three time points (months 1, 3, and 6 after enrollment) were presented and compared between HIV-negative and -positive partners using Chi-square tests for categorical variables and ANOVA for continuous variables. We conducted an exploratory factor analysis (EFA) to examine the structure of the behavioral modeling scale, using data from the first follow-up visit (e.g., 1 month after enrollment). The rationale for using factor analysis was based on this being a novel scale without previous exploration of its structure in this specific cultural and study setting. We used the promax factor rotation method that allows factor correlations and results in simple and interpretable factor structure [19]. The final factor structure was determined after an evaluation of eigenvalues, residual variances, chi-square, Bayesian Information Criterion (BIC), Comparative Fit Index (CFI), Root Mean Square Error of Approximation (RMSEA), and variance accounted for by different factor structures [20]. The internal consistency of the scale is indicated by Cronbach’s α. The data from both HIV-positive and negative partners were combined to conduct EFA and examine the internal consistency of the scale.

We examined the association between a participant’s behavioral modeling scale and subscales and their own self-reported PrEP/ART adherence score across three time points using generalized estimating equations (GEE) to account for repeated measurements. The regression coefficients and 95% confidence interval (CI) from the GEE model indicated the strength of the associations and the uncertainty of the estimates. We used logistic regression to examine the associations between the behavioral modeling scale and (a) outcome of viral suppression (HIV RNA < 1000 copies/ml) at 6 months post ART initiation and, in separate models, (b) outcome of TFV levels associated with high adherence (TFV > 700 fmol/punch) [21]. The odds ratio and 95%CI indicated the strength and precision of observed associations. Since we had only one time point with viral load and TVF outcome, behavioral model scale scores were averaged across month 1, 3, and 6 for this analysis. We also examined associations between behavioral modeling scale score and continuous TFV levels and viral load outcomes using linear regression. For all analyses, adjusted models were generated including participant age (continuous) and sex (male, female) as *a priori* determined confounding factors.

#### Ethics

The protocol was approved by ethics committees at the Uganda National HIV/AIDS Research Committee (ARC 194), the Uganda National Council for Science and Technology (HS 2381), and the University of Washington Human Subjects Division (STUDY00000320). The Kampala Capital City Authority and Wakiso District gave permission for data to be abstracted from clinic medical records. All participants for research procedures underwent an informed consent process and provided written consent in their preferred language.

## Results

### Descriptive Characteristics

In total, both members of 149 serodifferent couples enrolled for the research cohort at baseline, with 141 retained at month 1, 3 and 139 at month 6. More than half of the partners living with HIV were female (64%). The median age of the participants was 28 years (IQR 9.8). Based on self-report adherence score, the partners living with HIV were more adherent to ART than their partners were to PrEP, across all three follow-up visits (Table 1).

**Table 1:** Baseline characteristics of HIV-serodifferent couples

	HIV Negative Partner (*N* = 149)	HIV Positive Partner (*N* = 149)	Total (*N* = 298)	Test statistics	*p*
Age (years)				2.11	0.035^a^
Median (IQR)	29 (8.0)	27 (9.0)	28 (9.8)		
Gender				23.68	< 0.001^b^
Female	53 (35.6%)	96 (64.4%)	149 (50.0%)		
Virally suppressed^c^	NA	101 (86.3%)	NA	NA	
TFV detectable^d^	19 (59.4%)	NA	NA		
Month 1	*N* = 141	*N* = 141	*N* = 282		
Partner took ART/PrEP				5.27	0.007^b^
Yes	134 (95.0%)	141 (100.0%)	275 (97.5%)		
Partner took ART/PrEP properly				-1.08	0.249^b^
Very poor	1 (0.7%)	0 (0.0%)	1 (0.4%)		
Fair	3 (2.2%)	0 (0.0%)	3 (1.1%)		
Good	13 (9.7%)	10 (7.1%)	23 (8.4%)		
Very Good	49 (36.6%)	60 (42.6%)	109 (39.6%)		
Excellent	68 (50.7%)	71 (50.4%)	139 (50.5%)		
Partner took ART/PrEP often				-2.61	0.026^b^
Some Days	4 (3.0%)	0 (0.0%)	4 (1.5%)		
Most Days	20 (14.9%)	12 (8.5%)	32 (11.6%)		
Everyday	110 (82.1%)	129 (91.5%)	239 (86.9%)		
Partner adherence score				-3.94	< 0.001^a^
Mean (SD)	94.8 (8.4)	97.9 (3.7)	96.4 (6.6)		
Month 3	*N* = 141	*N* = 141	*N* = 282		
Partner took ART/PrEP				9.01	0.001^b^
Yes	128 (90.8%)	139 (99.3%)	267 (95.0%)		
Partner took ART/PrEP properly				-0.50	0.587^b^
Very poor	0 (0.0%)	1 (0.7%)	1 (0.4%)		
Poor	1 (0.8%)	0 (0.0%)	1 (0.4%)		
Fair	1 (0.8%)	1 (0.7%)	2 (0.7%)		
Good	15 (11.7%)	10 (7.2%)	25 (9.4%)		
Very Good	50 (39.1%)	60 (43.2%)	110 (41.2%)		
Excellent	61 (47.7%)	67 (48.2%)	128 (47.9%)		
Partner took ART/PrEP often				-3.12	< 0.001^b^
Some Days	2 (1.6%)	4 (2.9%)	6 (2.2%)		
Most Days	35 (27.3%)	10 (7.2%)	45 (16.9%)		
Everyday	91 (71.1%)	125 (89.9%)	216 (80.9%)		
Missing	21	10	31		
Partner adherence score				-4.66	< 0.001^a^
Mean (SD)	92.4 (7.6)	96.8 (7.7)	94.7 (8.0)		
Month 6	*N* = 139	*N* = 139	*N* = 278		
Partner took ART/PrEP				29.03	< 0.001^b^
Yes	104 (75.4%)	139 (97.9%)	243 (86.8%)		
Partner took ART/PrEP properly				-1.06	0.482 ^b^
Poor	1 (1.0%)	1 (0.7%)	2 (0.8%)		
Fair	3 (2.9%)	1 (0.7%)	4 (1.6%)		
Good	12 (11.5%)	10 (7.2%)	22 (9.1%)		
Very Good	30 (28.8%)	47 (33.8%)	77 (31.7%)		
Excellent	58 (55.8%)	80 (57.6%)	138 (56.8%)		
Partner took ART/PrEP often				-1.65	0.231^b^
Some Days	4 (3.8%)	2 (1.4%)	6 (2.5%)		
Most Days	19 (18.3%)	18 (12.9%)	37 (15.2%)		
Everyday	81 (77.9%)	119 (85.6%)	200 (82.3%)		
Partner adherence score				-2.05	< 0.001^a^
Mean (SD)	93.5 (10.6)	96.0 (7.9)	95.0 (9.2)		

^a^T-test

^b^Chi-square Test

^c^Viral suppression definition: HIV RNA < 1000 copies/ml

^d^TFV detectable: TFV > 700 fmol/punch. Available data for TFV (*n* = 32)

### Exploratory Factor Analysis

The Kaiser-Meyer-Olkin index of sampling adequacy for the behavioral modeling scale was 0.92, suggesting the data were suitable for factor analysis. Eigenvalues from factor analysis with promax rotation suggested a 5-factor model would be the most explanatory, with the first five components having an eigenvalue larger than one and the five factors accounting for 66% of the total variance (Table 2). As the number of factors increased from three to seven, Chi-square statistics and RMSEA both decreased, while CFI increased, suggesting improvement in model performance. Model fit index BIC decreased initially as the number of factors increased from three to five, suggesting improvement in model performance. BIC started to increase after the factor number exceeded five, suggesting that the model performance started to decrease. The fit statistics together supported the five-factor model. Additionally, the five-factor model provided a meaningful interpretation of the factor structure based on the factor loadings (Table 3). The five factors were: (1) attention to partner behavior, (2) collective action, (3) partner as a role model, (4) motivation, and (5) relationship quality. The mean and SD of factor correlations were presented in Table 4. When we split the dataset by HIV status, the factor structure was replicated in both subgroups (Supplement Tables 1, 2 and 3).

**Table 2:** Fit statistics of explorative factor analysis models

Number of factors	Chi-square statistics	*p*	BIC	CFI	RMSEA	RMSEA (lowr)	RMSEA (upper)	Variance accounted
3	446.546	< 0.001	-254.896	0.845	0.115	0.108	0.123	0.580
4	234.341	< 0.002	-412.747	0.903	0.096	0.088	0.105	0.626
5	135.689	< 0.003	-448.326	0.932	0.085	0.077	0.095	0.660
6	85.404	< 0.004	-446.415	0.951	0.077	0.067	0.087	0.689
7	44.050	< 0.005	-460.555	0.972	0.062	0.050	0.073	0.712

**Table 3:** Factor loadings of five-factor model

	Factor 1	Factor 2	Factor 3	Factor 4	Factor 5
Factors	Relationship	Attention	Role modeling	Motivation	Collective Action
**Proportion of variance explained (%)**	20.120	15.210	12.520	9.110	9.070
**Factor 1. Relationship**					
1. I feel close to my partner	1.030	-0.049	-0.166	-0.008	-0.033
2. I support my partner	0.869	-0.069	-0.046	0.046	0.040
3. I communicate will with my partner.	0.872	0.027	0.032	-0.008	-0.066
4. I get along well with my partner.	0.913	0.058	-0.040	-0.013	-0.072
5. My partner and I have a good relationship.	0.905	0.008	-0.047	0.006	0.010
6. When my partner takes PrEP I have hope for our future together.	0.481	0.014	0.141	0.022	0.177
7. I feel supported when my partner takes PrEP.	0.520	0.041	0.292	-0.032	0.023
**Factor 2. Attention**					
8. I often see my partner taking PrEP.	0.055	0.796	-0.060	-0.025	0.176
9. Most days, I see my partner taking PrEP.	-0.037	0.897	-0.023	-0.021	0.165
10. I have watched my partner become good at taking PrEP.	0.013	0.908	0.098	-0.092	-0.006
11. I have watched my partner struggle to take PrEP.	-0.028	0.773	0.035	0.056	-0.034
12. I know my partner takes PrEP regularly.	0.027	0.491	0.415	0.069	-0.189
**Factor 3. Role modeling**					
13. My partner is good at taking PrEP.	-0.019	0.043	0.878	0.010	-0.110
14. My partner takes PrEP the way it is supposed to be taken.	-0.005	0.178	0.736	0.058	-0.130
**Factor 4. Motivation**					
15. My partner has no side effects from PrEP.	0.099	-0.018	-0.119	0.932	-0.008
16. My partner feels good taking PrEP.	0.125	-0.010	0.214	0.593	0.017
17. PrEP is working out well for my partner.	0.102	0.038	0.399	0.462	-0.046
18. No bad things have happened because of my partner taking PrEP.	-0.066	0.004	-0.033	0.612	0.052
19. My partner is more healthy because they take PrEP.	-0.171	-0.104	0.484	0.354	0.100
**Factor 5. Collective action**					
20. I remind my partner to take PrEP.	0.043	-0.155	0.464	-0.114	0.617
21. I encourage my partner to take PrEP each day.	0.033	-0.106	0.520	-0.124	0.550
22. I provide my partner with material support to take PrEP every day. Material support includes pouring a glass of water, providing a meal, or providing money for a meal for swallowing PrEP.	-0.044	0.310	0.061	-0.074	0.546
23. I take my ART at the same time of day my partner takes PrEP.	-0.031	-0.005	-0.201	0.095	0.678
24. I use my partner’s PrEP reminder tools to remember to take my ART. Reminder tools might include phone alarm, radio, or a tablet box.	0.028	0.254	-0.152	0.117	0.581

**Table 4:** Factor correlations

	Factor 1	Factor 2	Factor 3	Factor 4	Factor 5
	Relationship	Attention	Role modeling	Motivation	Collective action
1	2.5 (0.54)^a^	0.571	0.652	0.408	0.461
2	-	2.5 (0.65)	0.629	0.445	0.602
3	-	-	2.6 (0.52)	0.639	0.572
4	-	-	-	2.4 (0.52)	0.336
5	-	-	-	-	2 (0.68)

^a^Means (standard deviations) are presented on the diagonal cells

### Internal Consistency

The scale demonstrated a high level of internal consistency (Cronbach’s α = 0.93, 95% confidence interval [CI]: 0.92–0.95). The five subscales also demonstrated high internal consistency: relationship quality (α = 0.93, 95% CI: 0.92–0.94), attention to partner behavior (α = 0.92, 95% CI: 0.91–0.93), partner as a role model (α = 0.85, 95% CI: 0.81–0.89), motivation (α = 0.83, 95% CI: 0.80–0.87), and collective action (α = 0.80, 95% CI: 0.76–0.84). The internal consistency of the scale was similar between partners living with and without HIV (Supplement Table 4).

### Behavioral Modeling, Adherence Score, and Viral Suppression

The mean of the total score for our 24-item behavioral modeling measure was 57.2 (SD = 11.0) at month 1, 56.3 (SD = 12.6) at month 3, and 56.4 (SD = 2.5) at month 6, with no statistically significant difference between partners of the same couple. Compared to partners living with HIV, HIV-negative partners reported higher scores on motivation and role modeling subscales at 1-month (*t*(280) = 3.34, *p* < 0.001; *t*(180) = 2.74, *p* = 0.006) and 3-month follow-up visits (*t*(278) = 4.69, *p* < 0.001; *t*(278) = 3.26, *p* = 0.001). Compared to partners living with HIV, HIV-negative partners reported lower scores on relationship subscale (*t*(280) = -2.99, *p* = 0.005) and collective action (*t*(280) = -2.27, *p* = 0.026) at 6-month follow-up visit (Table 5).

**Table 5:** Behavioral modeling score at all visits by HIV status, mean (SD)

	HIV Negative Partner	HIV Positive Partner	Total	Test Statistics	*p* ^a^
Month 1	*N* = 142	*N* = 140			
Behavioral Modelling Scale (total score)	57.7 (10.7)	56.7 (11.3)	57.2 (11.0)	0.66	0.510
Relationship	17.4 (4.1)	17.7 (3.4)	17.5 (3.7)	-0.64	0.525
Motivation	12.1 (2.4)	11.0 (3.0)	11.6 (2.8)	3.34	< 0.001
Attention	12.4 (3.2)	12.4 (3.7)	12.4 (3.4)	0.06	0.951
Collective Action	10.0 (3.2)	9.8 (3.5)	9.9 (3.4)	0.43	0.668
Role Modeling	5.3 (1.0)	4.9 (1.4)	5.1 (1.2)	2.74	0.006
Month 3	*N* = 141	*N* = 139			
Behavioral Modelling Scale (total score)	57.5 (12.4)	55.0 (12.7)	56.3 (12.6)	1.65	0.100
Relationship	17.1 (4.5)	17.0 (3.9)	17.1 (4.2)	0.10	0.922
Motivation	12.9 (2.3)	11.4 (2.9)	12.2 (2.7)	4.69	< 0.001
Attention	12.5 (3.4)	12.2 (3.6)	12.3 (3.5)	0.77	0.441
Collective Action	9.7 (3.5)	9.6 (3.5)	9.7 (3.5)	0.38	0.706
Role Modeling	5.3 (1.0)	4.8 (1.3)	5.1 (1.2)	3.26	0.001
Month 6	*N* = 139	*N* = 143			
Behavioral Modelling Scale (total score)	55.3 (13.7)	57.8 (10.6)	56.4 (12.5)	-1.55	0.135
Relationship	16.5 (5.3)	18.1 (3.2)	17.2 (4.5)	-2.99	0.005
Motivation	12.7 (2.3)	12.1 (2.3)	12.4 (2.3)	1.93	0.055
Attention	12.0 (3.6)	12.6 (2.9)	12.3 (3.3)	-1.47	0.154
Collective Action	8.9 (3.7)	10.0 (3.3)	9.4 (3.6)	-2.27	0.026
Role Modeling	5.3 (1.0)	5.0 (1.1)	5.1 (1.1)	1.96	0.050
Across all visits	*N* = 129	*N* = 118			
Behavioral Modelling Scale (total score)	56.8 (12.3)	56.4 (11.7)	56.6 (12.0)	0.49	0.623
Relationship	17.0 (4.6)	17.6 (3.5)	17.2 (4.2)	-2.01	0.049
Motivation	12.6 (2.4)	11.4 (2.8)	12.0 (2.6)	5.87	< 0.001
Attention	12.3 (3.4)	12.4 (3.4)	12.3 (3.4)	-0.27	0.784
Collective Action	9.6 (3.5)	9.8 (3.4)	9.7 (3.5)	-0.84	0.403
Role Modeling	5.3 (1.0)	4.9 (1.3)	5.1 (1.2)	4.65	< 0.001

^a^T-test

HIV-negative partners’ behavior modeling of PrEP taking, indicated by the composite behavioral modeling scale score reported by HIV-positive partners, was positively associated with their HIV-positive partners’ ART adherence (*β* = 0.06, 95% CI: 0.01–0.10) among partners living with HIV, after accounting for age, and sex. In separate GEE models, subscales of motivation (*β* = 0.18, 95% CI: 0.02–0.38), relationship quality (*β* = 0.19, 95% CI: 0.02–0.33), attention (*β* = 0.18, 95% CI: 0.02–0.35), and role modeling (*β* = 0.50, 95% CI: 0.07–0.93) were positively associated with self-reported ART adherence score (Table 6).

**Table 6:** Associations between behavioral modeling scores and self-reported ART adherence score (partners living with HIV)

			Unadjusted			Adjusted^b^		
Behavioral modeling score^a^	*N*	Observations	*β*	*CI*	*p*	*β*	*CI*	*p*
MB Total	139	346	0.06	0.02–0.11	**0.006**	0.06	0.01–0.10	**0.017**
Motivation	141	358	0.21	0.01–0.41	**0.007**	0.18	0.02–0.38	**0.020**
Relationship	139	346	0.2	0.05–0.36	**0.009**	0.17	0.02–0.33	**0.019**
Attention	141	358	0.2	0.04–0.36	**0.030**	0.18	0.02–0.35	**0.045**
Collective Action	141	358	0.11	-0.05–0.27	0.113	0.09	-0.07–0.25	0.189
Role Modeling	141	358	0.56	0.12–0.99	**0.018**	0.5	0.07–0.93	**0.028**

^a^Each row is a separate model. All models were GEE. The main predictor of each model is listed in the table rows, the outcome of all models was self-reported ART adherence score

^b^The adjusted models controlled for age and gender

HIV-positive partners’ behavioral modeling of ART taking, indicated by the composite behavioral modeling scale score reported by HIV-negative partners, was positively associated with their self-reported PrEP adherence (*β* = 0.25, 95% CI: 0.17–0.33) after controlling for age and sex. In separate GEE models, subscales for motivation (*β* = 1.09, 95% CI: 0.71–1.47), relationship quality (*β* = 0.56, 95% CI: 0.34–0.78), attention (*β* = 0.69, 95% CI: 0.39–0.99), collective action (*β* = 0.64, 95% CI: 0.35–0.93), and role modeling (*β* = 2.76, 95% CI: 1.87–3.66) were positively associated with ART adherence score (Table 7).

**Table 7:** GEE models: behavioral modeling and self-reported PrEP adherence score (HIV-negative partners)

			Unadjusted			Adjusted^b^		
Models^a^	*N*	Observations	*β*	*CI*	*p*	*β*	*CI*	*p*
MB Total	138	350	0.25	0.17–0.33	< 0.001	0.25	0.17–0.33	< 0.001
Motivation	140	362	1.11	0.73–1.48	< 0.001	1.09	0.71–1.47	< 0.001
Relationship	138	350	0.56	0.35–0.78	< 0.001	0.56	0.34–0.78	< 0.001
Attention	140	362	0.71	0.41–1.00	< 0.001	0.69	0.39–0.99	< 0.001
Collective Action	140	361	0.65	0.36–0.94	< 0.001	0.64	0.35–0.93	< 0.001
Role Modeling	140	362	2.8	1.92–3.69	< 0.001	2.76	1.87–3.66	< 0.001

^a^Each row is a separate model. The main predictor of each model is listed in the table

^b^The adjusted models controlled for age and gender

When averaged across three follow-up visits, neither the behavioral modeling scale composite score, nor subscale scores were associated with either dichotomized or continuous parameterizations of viral load or TFV levels at the month 6 follow-up visit (Tables 8, 9, 10 and 11). The results were similar when limited to data from the 6-month time point. Self-reported ART adherence mapped to viral load data with 91.0% of people who took ART every day having suppressed viremia, a greater frequency than those who reported gaps in taking ART. Similarly, self-reported PrEP use was consistent with TFV level data, with 66.7% of people who took PrEP every day having detectable TFV levels, which was more frequent than people who self-reported low adherence to PrEP (Tables 12 and 13).

**Table 8:** Associations between modeling behavior and HIV viral load (continuous)

		Unadjusted			Adjusted^b^		
Models^a^	*N*	*β*	*CI*	*p*	*β*	*CI*	*p*
MB Total	108	-142.64	-369.16–83.87	0.215	-123.81	-356.23–108.62	0.293
Motivation	82	-60.74	-1355.45–1233.97	0.926	51.42	-1308.16–1410.99	0.940
Relationship	62	-314.28	-1203.74–575.17	0.482	-79.15	-1039.67–881.38	0.870
Attention	73	-536.56	-1559.08–485.95	0.299	-486.42	-1504.03–531.18	0.344
Collective Action	110	138.67	-626.35–903.70	0.720	250.18	-529.04–1029.41	0.526
Role Modeling	47	-9644.89	-16852.80 – -2436.99	0.010	-9082.44	-16733.97 – -1430.90	0.021

^a^Each row is a separate model. The main predictor of each model is listed in the table

^b^The adjusted models controlled for age, gender

**Table 9:** Association between modeling behavior and viral suppression (binary, HIV RNA < 1000 copies/ml)

		Unadjusted			Adjusted^b^		
Models^a^	*N*	*Odds Ratio*	*CI*	*p*	*Odds Ratio*	*CI*	*p*
MB Total	93	0.99	0.93–1.04	0.788	0.99	0.93–1.05	0.836
Motivation	99	1.01	0.81–1.24	0.916	1.01	0.80–1.24	0.921
Relationship	63	1.01	0.80–1.22	0.933	0.99	0.79–1.21	0.961
Attention	80	1	0.83–1.16	0.981	1	0.83–1.16	0.992
Collective Action	99	1.07	0.89–1.29	0.448	1.08	0.89–1.30	0.444
Role Modeling	69	0.71	0.33–1.18	0.286	0.72	0.33–1.19	0.295

^a^Each row is a separate model. The main predictor of each model is listed in the table

^b^The adjusted models controlled for age, gender

**Table 10:** Association between modeling behavior and TFV diphosphate levels (continuous)

		Unadjusted			Adjusted^b^		
Models^a^	*N*	*β*	*CI*	*p*	*β*	*CI*	*p*
MB Total	26	4.55	-44.71–53.80	0.851	6.29	-46.69–59.26	0.808
Motivation	13	264.24	-75.34–603.82	0.115	265.72	-121.32–652.75	0.155
Relationship	13	-0.4	-253.79–253.00	0.997	-59.91	-437.37–317.55	0.728
Attention	16	93.62	-137.79–325.03	0.4	238.4	-31.79–508.58	0.079
Collective Action	28	29.28	-103.81–162.37	0.655	30.59	-105.10–166.28	0.646
Role Modeling	10	298.64	-294.98–892.26	0.279	296.01	-451.75–1043.78	0.370

^a^Each row is a separate model. The main predictor of each model is listed in the table

^b^The adjusted models controlled for age, gender

**Table 11:** Association between modeling behavior and TFV detection (binary, TFV > 700 fmol/punch)

		Unadjusted			Adjusted^b^		
Models^a^	*N*	*Odds Ratio*	*CI*	*p*	*Odds Ratio*	*CI*	*p*
MB Total	26	1	0.86–1.15	0.958	0.45	0.07–2.70	0.384
Motivation	13	7.7	1.10–3123.54	0.274	9.98	1.10–27868.00	0.363
Relationship	13	0.99	0.50–1.86	0.962	0.78	0.26–1.82	0.575
Attention	16	2.23	0.87 – NA	0.688	4.22	1.24 – NA	0.287
Collective Action	28	1.05	0.73–1.51	0.764	1.06	0.73–1.52	0.732
Role Modeling	10	3.08	0.64–47.88	0.236	2.86	0.56–75.38	0.279

^a^Each row is a separate model. The main predictor of each model is listed in the table

^b^The adjusted models controlled for age, gender

**Table 12:** Concordance between ART adherence and viral suppression (HIV RNA < 1000 copies/ml)

	How often the client took their ART medication in the past month (Took ART often)			*k*
	Not everyday (*N* = 68)	Everyday (*N* = 81)	Total (*N* = 149)	
Viral suppression				0.12
No	10 (20.0%)	6 (9.0%)	16 (13.7%)	
Yes	40 (80.0%)	61 (91.0%)	101 (86.3%)	
	How well the client took their ART/PrEP medication as directed in the past month? (Took ART properly)			
	Not Excellent (*N* = 91)	Excellent (*N* = 58)	Total (*N* = 149)	
Viral suppression				0.08
No	12 (17.6%)	4 (8.2%)	16 (13.7%)	
Yes	56 (82.4%)	45 (91.8%)	101 (86.3%)	

*Note* Thresholds for Cohen’s kappa: poor (< 0.2), fair (0.2–0.4), good (0.61–0.80), very good (0.081 − 1.00)

**Table 13:** Concordance between PrEP adherence and TFV detection (TFV > 700 fmol/punch)

	How often the client took their PrEP medication in the past month (Took PrEP often)			*k*
	Not Everyday (*N* = 8)	Everyday (*N* = 24)	Total (*N* = 32)	
TFV level				0.24
No	5 (62.5%)	8 (33.3%)	13 (40.6%)	
Yes	3 (37.5%)	16 (66.7%)	19 (59.4%)	
	How well the client took their ART/PrEP medication as directed in the past month? (Took PrEP properly)			
	Not Everyday (*N* = 15)	Everyday (*N* = 17)	Total (*N* = 32)	
TFV level				0.11
No	7 (46.7%)	6 (35.3%)	13 (40.6%)	
Yes	8 (53.3%)	11 (64.7%)	19 (59.4%)	

*Note* Thresholds for Cohen’s kappa (*k*): poor (< 0.2), fair (0.2–0.4), good (0.61–0.80), very good (0.081 − 1.00)

## Discussion

To our knowledge, this is the first study that developed a scale to measure dyadic behavioral modeling within HIV-serodifferent couples. We found that modeled ART-taking behavior was associated with PrEP use and that modeled PrEP-taking behavior was associated with ART use. This association was bidirectional irrespective of sex. The role model subscale was positively associated with self-reported adherence to ART and PrEP.

The final factor structure of the scale (attention, collective action, motivation, relationship, and role model) mapped well onto the four processes of observational learning. Notably, we grouped the items that were intended to measure retention (item 13–15) and production (item 16–17) and named this factor “collective action”, representing the joint efforts of both partners to support each other in medication adherence. Collective action is a construct of unique value in the context of measuring modeled behavior within a couple. While social cognitive theory recognizes individuals’ ability to actively construct their environment to achieve their individual goals, it also emphasizes collective action, where a group of individuals work together to enact change that can benefit the group [9]. The collective action within the couple, such as reminding each other to take medication, serves the goals of maintaining optimal health for both partners and preserving the long-term relationship. Prior work also suggested that the shared experience of taking antiretroviral medication together which often involves mutual reminders, and providing emotional and material support to each other, has been documented to draw members of the couples closer to each other and prevent their separation, contributing to the success of integrated delivery of ART and PrEP to HIV serodifferent couples [22].

Additionally, the measure included a “partner as a role model” factor that reflected a partner’s medication adherence behavior. Although this factor is composed of only two items, the role model subscale consistently predicted the adherence score of ART/PrEP and had the largest coefficient among all subscales. This is consistent with the literature on role modeling and behavior change. A study using peers as role models who were similar to participants along multiple dimensions (e.g., sex, age, occupation) found that the peer modeling intervention increased participants’ physical fitness, compared to the group that only received general health information [23]. A review of peer-based interventions on HIV-related outcomes found that peer-based interventions increased linkage and retention to HIV care [24]. The improved health behaviors might be due to the positive effect of exposure to peer models on participant confidence and knowledge. The behaviors of the partner might contribute to the establishment of a norm within the couple that is mutually reinforcing the adherent behavior [25].

When applied in clinical settings and research, our novel behavioral modeling scale can be used to outline intervention targets across five dimensions (motivation, relationship, attention, collective action, role modeling) for members of serodifferent couples to improve medication adherence and HIV prevention and treatment outcomes. This is consistent with the perspectives of sero-different couples where the participants reported factors such as relationship quality and share protection that motivated them to adopt HIV prevention strategies coupled with viral control [8]. HIV-serodifferent couples are uniquely positioned where the sustained HIV viremic control through ART and maximizing prevention benefit of PrEP can be attained through collective medication-taking efforts from both partners. Intervention programs that strategically target the different dimensions of behavioral modeling between partners can create a sense of collective action and promote optimal adherence to antiretrovirals of both partners. This measure was developed for the specific context of serodifferent couples to capture dimensions of dyadic behavioral modeling that are associated with adherence behavior in HIV prevention and treatment. However, this measure has the potential to be adapted and generalized to the measurement of dyadic behavioral modeling effect in other health-related research. Further studies are needed to examine the psychometric properties of this measure in other health behavior settings and cultural contexts.

This study has several limitations. First, we were not able to utilize all three self-reported adherence items when calculating the adherence scores due to the adaption of the first item in this study. Future studies may use the original adherence items to test the predictive effects of this behavioral modeling. Second, despite the concordance between self-reported adherence data and biological outcomes (viral suppression and TFV level), our data are insufficiently powered to determine whether the scale and subscales are associated with objective adherence measures, viral suppression and TFV. Future studies can collect a larger sample of biological outcomes and examine whether behavioral modeling has differential predictive effects for self-reported versus biological outcomes.

## Conclusions

Our novel behavioral modeling measure successfully assessed five dimensions of modeled behavior (i.e., attention, motivation, relationship quality, collective action, and role modeling), which are components of observational learning. Furthermore, our results suggested the behavioral modeling measure was associated with self-reported medication-adherence behavior among members of HIV-serodifferent couples. With further validation, this measure could be applied in clinical and research settings to outline modifiable behavioral change within partners using the five dimensions of behavioral modeling of medication taking. Furthermore, the results suggest that further interventions with HIV-serodifferent couples could think beyond role modeling, and include dimensions such as collective action and relationship quality between couples, to maximize adherence to antiretrovirals and protection against HIV transmission.

### Electronic Supplementary Material

Below is the link to the electronic supplementary material.

Supplementary Material 1

## Data Availability

Data and material will be available upon request. Please contact icrc@uw.edu with any requests.

## Code Availability (Software Application or Custom Code)

Code will be available upon request. Please contact icrc@uw.edu to request code.
