# Supplementary material for: Behavioral Modeling and its Association with PrEP and ART Use in Ugandan HIV-Serodifferent Couples
Source: AIDS Behav. 2024 Feb 16;28(5):1719–30. doi: 10.1007/s10461-024-04286-2 (PMC11069469; doi:10.1007/s10461-024-04286-2)
Supplement: Supplementary file 1 — Supplementary Material 1 [file 10461_2024_4286_MOESM1_ESM.docx]

**Table 1.** Original questionnaire with 28 questions

| Relationship quality |
| --- |
| I feel close to my partner  My partner and I are a lot alike.  I have a lot in common with my partner.  I tend to follow my partner’s example.  My partner is a role model to me in many ways.  I think I am a role model for my partner  In general, I do what my partner does.  My partner supports me.  I support my partner. |
| ATTENTION |
| I often see my partner taking PrEP  Most days, I see my partner taking PrEP.  I have watched my partner become good at taking PrEP.  I have watched my partner struggle to take PrEP.  I know my partner takes PrEP regularly. |
| RETENTION |
| I remind my partner to take PrEP.  I encourage my partner to take PrEP each day.  I provide my partner with material support to take PrEP every day. Material support includes pouring a glass of water, providing a meal, or providing money for a meal for swallowing PrEP. |
| REPRODUCTION |
| I take my ART at the same time of day my partner takes PrEP.  I use my partner’s PrEP reminder tools to remember to take my ART. Reminder tools might include phone alarm, radio, or a pill box |
| MOTIVATION |
| When my partner takes PrEP I have hope for our future together.  I feel supported when my partner takes PrEP.  My partner has no side effects from PrEP.  My partner feels good taking PrEP.  PrEP is working out well for my partner.  My partner takes PrEP to stay healthy.  My partner is good at taking PrEP.  My partner takes PrEP the way it is supposed to be taken.  No bad things have happened because of my partner taking PrEP. |

**Table 2.** Factor Loadings of Five-Factor Model for Partners Living with HIV

|  | **Factor 1** | **Factor 2** | **Factor 3** | **Factor 4** | **Factor 5** |
| --- | --- | --- | --- | --- | --- |
|  | **Relationship** | **Attention** | **Role modeling** | **Motivation** | **Collective Action** |
| **Proportion of variance explained (%)** | **0.200** | **0.150** | **0.130** | **0.090** | **0.110** |
| **Factor 1. Relationship** |  |  |  |  |  |
| 1. I feel close to my partner | **1.035** | -0.072 | -0.144 | -0.058 | -0.035 |
| 2. I support my partner | **0.838** | -0.094 | -0.081 | 0.112 | 0.002 |
| 3. I communicate will with my partner. | **0.843** | -0.077 | 0.163 | -0.079 | 0.022 |
| 4. I get along well with my partner. | **0.888** | 0.149 | -0.111 | -0.034 | -0.047 |
| 5. My partner and I have a good relationship. | **0.962** | 0.002 | -0.035 | -0.094 | 0.032 |
| 6. When my partner takes PrEP I have hope for our future together. | **0.433** | 0.037 | 0.051 | 0.125 | 0.126 |
| 7. I feel supported when my partner takes PrEP. | **0.443** | 0.043 | 0.312 | 0.010 | -0.026 |
| **Factor 2. Attention** |  |  |  |  |  |
| 8. I often see my partner taking PrEP. | 0.106 | **0.792** | -0.143 | 0.026 | 0.170 |
| 9. Most days, I see my partner taking PrEP. | -0.002 | **0.895** | -0.052 | 0.064 | 0.081 |
| 10. I have watched my partner become good at taking PrEP. | 0.006 | **0.868** | 0.152 | -0.089 | 0.015 |
| 11. I have watched my partner struggle to take PrEP. | -0.140 | **0.813** | 0.087 | 0.117 | -0.123 |
| 12. I know my partner takes PrEP regularly. | 0.112 | **0.334** | 0.556 | -0.018 | -0.098 |
| **Factor 3. Role modeling** |  |  |  |  |  |
| 13. My partner is good at taking PrEP. | -0.027 | 0.015 | **1.034** | -0.197 | 0.005 |
| 14. My partner takes PrEP the way it is supposed to be taken. | -0.041 | 0.236 | **0.736** | -0.046 | 0.011 |
| **Factor 4. Motivation** |  |  |  |  |  |
| 15. My partner has no side effects from PrEP. | 0.032 | 0.058 | 0.001 | **0.952** | -0.193 |
| 16. My partner feels good taking PrEP. | 0.239 | -0.030 | 0.173 | **0.559** | 0.031 |
| 17. PrEP is working out well for my partner. | 0.207 | -0.006 | 0.462 | **0.380** | -0.063 |
| 18. No bad things have happened because of my partner taking PrEP. | -0.170 | -0.152 | 0.504 | **0.276** | 0.185 |
| 19. My partner is more healthy because they take PrEP. | -0.095 | 0.071 | -0.190 | **0.740** | 0.085 |
| **Factor 5. Collective action** |  |  |  |  |  |
| 20. I remind my partner to take PrEP. | 0.004 | -0.007 | 0.090 | -0.062 | **0.862** |
| 21. I encourage my partner to take PrEP each day. | -0.043 | -0.071 | 0.296 | -0.019 | **0.704** |
| 22. I provide my partner with material support to take PrEP every day. Material support includes pouring a glass of water, providing a meal, or providing money for a meal for swallowing PrEP. | 0.019 | 0.160 | 0.080 | -0.120 | **0.661** |
| 23. I take my ART at the same time of day my partner takes PrEP. | 0.007 | -0.068 | -0.137 | 0.036 | **0.653** |
| 24. I use my partner's PrEP reminder tools to remember to take my ART. Reminder tools might include phone alarm, radio, or a tablet box. | 0.032 | 0.257 | -0.043 | 0.079 | **0.542** |

**Table 3**. Factor Loadings of Five-Factor Model for HIV-negative Partners

|  | **Factor 1** | **Factor 2** | **Factor 3** | **Factor 4** | **Factor 5** |
| --- | --- | --- | --- | --- | --- |
|  | **Relationship** | **Attention** | **Role modeling** | **Motivation** | **Collective Action** |
| **Proportion of variance explained (%)** | **0.310** | **0.240** | **0.180** | **0.137** | **0.134** |
| **Factor 1. Relationship** |  |  |  |  |  |
| 1. I feel close to my partner | **0.959** | -0.036 | -0.078 | 0.073 | -0.011 |
| 2. I support my partner | **0.782** | -0.027 | 0.134 | -0.003 | 0.006 |
| 3. I communicate will with my partner. | **0.997** | 0.134 | -0.173 | 0.059 | -0.110 |
| 4. I get along well with my partner. | **0.930** | 0.044 | -0.027 | 0.038 | -0.078 |
| 5. My partner and I have a good relationship. | **0.778** | -0.014 | 0.094 | 0.041 | 0.042 |
| 6. When my partner takes PrEP I have hope for our future together. | **0.309** | -0.035 | 0.470 | -0.071 | 0.170 |
| 7. I feel supported when my partner takes PrEP. | **0.376** | 0.013 | 0.565 | -0.097 | -0.016 |
| **Factor 2. Attention** |  |  |  |  |  |
| 8. I often see my partner taking PrEP. | -0.022 | **0.790** | 0.028 | -0.055 | 0.186 |
| 9. Most days, I see my partner taking PrEP. | -0.091 | **0.884** | 0.008 | -0.082 | 0.226 |
| 10. I have watched my partner become good at taking PrEP. | 0.045 | **0.944** | -0.004 | -0.057 | -0.021 |
| 11. I have watched my partner struggle to take PrEP. | 0.051 | **0.781** | -0.049 | 0.037 | 0.014 |
| 12. I know my partner takes PrEP regularly. | 0.081 | **0.632** | 0.141 | 0.099 | -0.223 |
| **Factor 3. Role modeling** |  |  |  |  |  |
| 13. My partner is good at taking PrEP. | 0.021 | 0.004 | **0.782** | 0.150 | -0.189 |
| 14. My partner takes PrEP the way it is supposed to be taken. | 0.044 | 0.089 | **0.740** | 0.133 | -0.270 |
| **Factor 4. Motivation** |  |  |  |  |  |
| 15. My partner has no side effects from PrEP. | 0.125 | -0.075 | -0.115 | **0.844** | 0.143 |
| 16. My partner feels good taking PrEP. | 0.023 | -0.003 | 0.248 | **0.599** | 0.072 |
| 17. PrEP is working out well for my partner. | -0.052 | 0.020 | 0.485 | **0.454** | 0.022 |
| 18. No bad things have happened because of my partner taking PrEP. | -0.120 | -0.020 | 0.343 | **0.468** | 0.094 |
| 19. My partner is more healthy because they take PrEP. | 0.038 | 0.020 | -0.089 | **0.576** | 0.023 |
| **Factor 5. Collective action** |  |  |  |  |  |
| 20. I remind my partner to take PrEP. | 0.294 | -0.105 | 0.285 | -0.087 | **0.397** |
| 21. I encourage my partner to take PrEP each day. | 0.287 | 0.000 | 0.305 | -0.161 | **0.383** |
| 22. I provide my partner with material support to take PrEP every day. Material support includes pouring a glass of water, providing a meal, or providing money for a meal for swallowing PrEP. | -0.034 | 0.363 | -0.030 | -0.014 | **0.528** |
| 23. I take my ART at the same time of day my partner takes PrEP. | -0.012 | -0.051 | -0.255 | 0.115 | **0.790** |
| 24. I use my partner's PrEP reminder tools to remember to take my ART. Reminder tools might include phone alarm, radio, or a tablet box. | -0.045 | 0.114 | -0.080 | 0.117 | **0.718** |

**Table 4.** Factor Correlations

|  | **Partner living with HIV** | | | | | **HIV-negative partner** | | | | |
| --- | --- | --- | --- | --- | --- | --- | --- | --- | --- | --- |
|  | **Factor 1** | **Factor 2** | **Factor 3** | **Factor 4** | **Factor 5** | **Factor 1** | **Factor 2** | **Factor 3** | **Factor 4** | **Factor 5** |
|  | **Relationship** | **Attention** | **Role modeling** | **Motivation** | **Collective Action** | **Relationship** | **Attention** | **Role modeling** | **Motivation** | **Collective Action** |
| 1 | 2.5 (0.48) | 0.594 | 0.643 | 0.384 | 0.558 | 2.5 (0.58) | 0.522 | 0.733 | 0.500 | 0.233 |
| 2 | - | 2.6 (0.65) | 0.718 | 0.654 | 0.565 | - | 2.5 (0.65) | 0.631 | 0.590 | 0.311 |
| 3 | - | - | 2.5 (0.58) | 0.645 | 0.723 | - | - | 2.7 (0.45) | 0.613 | 0.445 |
| 4 | - | - | - | 2.3 (0.56) | 0.540 | - | - | - | 2.5 (0.47) | 0.124 |
| 5 | - | - | - | - | 2 (0.7) | - | - | - | - | 2 (0.66) |

Note: Means (standard deviations) are presented on the diagonal cells.

**Table 5.** International Consistency of the Behavioral Modeling Measure

|  | **Partner living with HIV** | | | **HIV-negative partner** | | | **All participants** | | |
| --- | --- | --- | --- | --- | --- | --- | --- | --- | --- |
|  | *α* | *95%CI* | | *α* | *95%CI* | | *α* | *95%CI* | |
| Relationship | 0.92 | 0.89 | 0.94 | 0.94 | 0.93 | 0.96 | 0.93 | 0.92 | 0.94 |
| Attention | 0.92 | 0.90 | 0.94 | 0.92 | 0.90 | 0.94 | 0.92 | 0.91 | 0.93 |
| Role Modeling | 0.87 | 0.83 | 0.92 | 0.81 | 0.75 | 0.88 | 0.85 | 0.81 | 0.89 |
| Motivation | 0.85 | 0.81 | 0.89 | 0.80 | 0.75 | 0.86 | 0.83 | 0.80 | 0.87 |
| Collective Action | 0.84 | 0.79 | 0.88 | 0.78 | 0.72 | 0.84 | 0.80 | 0.76 | 0.84 |
| **Overall scale** | **0.94** | **0.93** | **0.96** | **0.93** | **0.91** | **0.95** | **0.93** | **0.92** | **0.95** |
